# Editorial: Machine learning for mining plant functional genes

**DOI:** 10.3389/fpls.2026.1795967

**Published:** 2026-02-25

**Authors:** Shanwen Sun, Quan Zou, Lijun Dou

**Affiliations:** 1 College of Life Science, Northeast Forestry University, Harbin, China; 2 Institute of Fundamental and Frontier Sciences, University of Electronic Science and Technology of China, Chengdu, China; 3 Cleveland Clinic Research, Cleveland Clinic, Cleveland, OH, United States

**Keywords:** large language models, machine learning, multi-omics integration, plant functional genomics, regulatory network inference

Plants in natural and agricultural environments are continuously challenged by diverse biotic and abiotic stresses, placing sustained pressure on crop productivity and global food security. The identification and functional characterization of plant genes underlying stress adaptation, development, and agronomic traits are therefore central to modern plant biology and precision breeding (Gaccione et al., 2025; Zhang et al., 2025). Although advances in high-throughput sequencing have generated vast genomic resources, experimentally validated functional annotations remain limited, leaving a substantial fraction of plant genes poorly characterized. A central challenge in contemporary plant genomics is thus to bridge the gap between rapidly expanding data volumes and biologically meaningful functional insights (Zhang et al., 2025). Recent progress in machine learning (ML) has created unprecedented opportunities to address this challenge. By integrating heterogeneous data types, spanning genome sequences, epigenomic marks, transcriptomic profiles, protein features, metabolite measurements, and regulatory interactions, ML-based approaches can model complex, nonlinear relationships that are difficult to resolve using conventional analytical frameworks (Sasse et al., 2024). Within this context, the Research Topic Machine Learning for Mining Plant Functional Genes brings together eight studies that collectively demonstrate how data-driven computational strategies are reshaping functional gene discovery, regulatory analysis, and trait dissection in plants.

A critical methodological shift in recent years is the emergence of foundation models (FMs) and large language models (LLMs) for biological sequence analysis. By conceptualizing DNA as a structured language, these models leverage large-scale pretraining to generate transferable representations that capture latent regulatory and functional features. Compared with task-specific models, FMs offer enhanced generalization, cross-species transferability, and scalability, making them particularly attractive for plant systems characterized by genomic complexity and limited functional annotations. Providing a systematic overview of this paradigm, Xu et al. present a mini-review synthesizing recent advances in foundation models for plant molecular biology. The review traces the evolution from general DNA language models to plant-specific tools and highlights key challenges unique to plant systems, including polyploidy, repetitive genomes, and sparse experimental annotations. By outlining future directions such as multimodal integration and computational efficiency, this work establishes a conceptual framework for understanding how FMs are redefining computational plant biology and guiding next-generation model development.

Building on this FM paradigm, several contributions demonstrate how representation learning can be applied to concrete biological problems. Zhang et al. applied a DNABERT-2–based framework combined with gradient boosting to identify DNA N6-methyladenine modifications in rice, illustrating how foundation models can enhance epigenetic marker detection while mitigating data sparsity. This work exemplifies a broader shift toward pretraining-based strategies in plant genomics, with implications for cross-species prediction and regulatory annotation.

Extending LLM-based approaches to cis-regulatory element discovery, Pu et al. developed an enhancer identification framework that couples DNABERT-2 feature extraction with a support vector machine classifier. Beyond predictive performance, this study addresses the interpretability challenge inherent to deep learning by introducing a differential entropy–based analysis to monitor class separation during fine-tuning. The results provide theoretical insight into training dynamics and offer a principled strategy for model optimization, highlighting the growing emphasis on transparency and interpretability in genomic ML.

In parallel with advances in representation learning, architectural innovation is driving progress in modeling gene expression and regulatory complexity. Guo et al. proposed a hybrid framework that integrates Transformer-based global attention with state space models to efficiently capture both long-range dependencies and local regulatory motifs. Validated across multiple crop species, this approach demonstrates improved accuracy and generalization over conventional convolutional architectures, underscoring the importance of model design choices in decoding plant regulatory syntax.

Beyond sequence-centric modeling, graph-based learning emerges as a powerful strategy for uncovering regulatory interactions mediated by non-coding RNAs. Addressing the sparsity and noise characteristic of experimental interaction data, Liao et al. introduced an interpretable graph representation learning framework for predicting plant RNA–RNA interactions. By combining robustness-enhancing masking strategies with biologically interpretable decoding, this work advances the analysis of post-transcriptional regulatory networks, particularly in the context of stress responses.

Several studies focus directly on agriculturally relevant traits and stress adaptation. Qiao et al. developed a machine learning framework for identifying saline–alkali tolerance genes, explicitly addressing data imbalance and sequence divergence through cost-sensitive learning and evolutionary feature extraction. Importantly, their analysis links predictive features to known physiological mechanisms, illustrating how ML can generate biologically interpretable insights rather than purely statistical predictions. Complementing this gene-centric perspective, Saavedra et al. applied interpretable ML to whole-genome methylation data to resolve dormancy stages in sweet cherry. By integrating ensemble learning with feature attribution analysis, the study identifies epigenetic markers associated with developmental transitions and agronomic traits, demonstrating the potential of ML-derived biomarkers for crop management.

Finally, Ju et al. exemplify the value of multi-omics integration by combining genome-wide association analysis with transcriptomic and metabolomic data to dissect seed germination mechanisms in sorghum. By linking genetic variation to hormone signaling and metabolic flux, this systems-level approach moves beyond association toward mechanistic understanding, reinforcing the importance of integrative frameworks in functional gene discovery.

Taken together, the contributions in this Research Topic highlight the transformative role of machine learning and foundation models in plant functional genomics. By advancing representation learning, model architecture, interpretability, and multi-omics integration, these studies move the field beyond traditional sequence-based annotation toward predictive, mechanism-aware, and application-oriented frameworks. Continued synergy between computational innovation and experimental validation will be essential for translating these advances into resilient, high-yield crops capable of meeting future agricultural and environmental challenges.
